# Green and chemical synthesis of Cu_₂_O nanoparticles for antibacterial functionalization of textiles: a comparative study against *Escherichia coli* strains

**DOI:** 10.1128/spectrum.03212-25

**Published:** 2026-04-03

**Authors:** Luis Castañeda Pelaez, David Asmat-Campos, Jesús Rojas-Jaimes, Gabriela Montes de Oca-Vásquez, Miguel Mogollón Almidón, Carlos Peralta Siesquen

**Affiliations:** 1 Facultad de Ciencias de la Salud, Universidad Privada del Norte 422527 , Los Olivos, Peru; 2 Dirección de Investigación, Innovación & Responsabilidad Social, Universidad Privada del Norte 422527 , Trujillo, Peru; 3 Centro de Investigación en Nanotecnología y Materiales Avanzados (CINMA), Universidad Privada del Norte 33220, Trujillo, Peru; 4 Dirección de Investigación, Innovación & Responsabilidad Social, Universidad Privada del Norte, Lima, Perú; 5 Escuela de Medicina Humana, Universidad Científica del Sur, Lima, Peru; 6 Laboratorio Nacional de Nanotecnología (LANOTEC), Centro Nacional de Alta Tecnología 541212, San José, Costa Rica; 7 Facultad de Ciencias de la Salud, Universidad Privada del Norte, Breña 33220, Lima, Peru; 8 Laboratorio de Microbiología, IPRESS Jorge Chávez, Puerto Maldonado, Peru; Seton Hall University, South Orange, New Jersey, USA

**Keywords:** green synthesis, biogenic synthesis, Cu_2_O nanoparticles, functionalized textiles

## Abstract

This study evaluates the synthesis of cuprous oxide (Cu₂O) nanoparticles via biogenic and chemical methods, employing *Myrciaria dubia* juice and ascorbic acid as reducing agents, respectively. The biogenic synthesis yielded nanoparticles with an average size of 10 ± 2.22 nm, whereas the chemical method produced particles averaging 75 ± 2.8 nm. The nanoparticles were incorporated into textiles (70% cotton, 30% polyester) through *in situ* and post-synthesis functionalization methods. The functionalized textiles exhibited significant antibacterial efficacy against *Escherichia coli* ATCC 25922, with inhibition rates exceeding 99.9%: 99.98% for the chemical post-synthesis method, 99.94% for the biogenic post-synthesis method, and 99.90% for the biogenic *in situ* method. However, no significant antibacterial effect was observed against extended-spectrum β-lactamase (ESBL)-producing *E. coli* strains. These findings highlight the critical role of nanoparticle size and functionalization method in antimicrobial effectiveness, underscoring the potential of green synthesis for the development of sustainable antibacterial textiles.

**IMPORTANCE:**

This article demonstrates a differential response to copper oxide nanoparticles between an antibiotic-sensitive *Escherichia coli* strain and another strain of *E. coli* resistant to antibiotics, such as extended-spectrum beta-lactamases. Therefore, it could be thought that resistance to the effects of copper oxide nanoparticles could be related to antibiotic resistance.

## INTRODUCTION

In recent years, research has focused on producing nanoparticles (NPs) using eco-friendly methods in which synthetic chemicals are replaced by organic compounds responsible for the precursor reduction process. The latter are economical and produce fewer polluting residues. Those “green” methods using extracts from leaves, fruits, stems, and barks of various plants are among the most widely used (1–3). The polyphenolic compounds, reducing sugars, and amino acids present in these extracts are indicated as those responsible for the process of reduction and formation of nanoparticles (4, 5).

Extracts from six plants, *Eucalyptus camaldulensis, Azadirachta indica, Murraya koenigii, Avicennia marina, Rosa rubiginosa,* and *Datura stramonium*, for example, were used as reducing agents in the synthesis process of CuO NPs, showing peaks of plasmon resonance between 563 and 582 nm in their UV-vis characterization, confirming nanoparticle sizes between 29 and 48 nm by transmission electron microscopy (TEM). Fourier transform infrared (FTIR) characterization reveals that biomolecules, such as proteins, polyphenols, and other phytochemical components present in the extracts, act as reducing agents (6). The effects of extract percentage, pH, and temperature were analyzed to obtain copper nanoparticles, finding that, at 100% extract concentration, the wavelength of the plasmon resonance peak is shorter than at other concentrations. This indicates that the particle sizes are smaller. As for the pH and temperature values, smaller nanoparticles are obtained for alkaline pH values and temperatures around 60°C (7).

Current reports show CuO NPs as potential bactericides and fungicides (8–10). Using eucalyptus (*Eucalyptus globulus L*.) and peppermint (*Mentha piperita*) leaf extract, CuO NPs were synthesized and characterized, obtaining sizes between 10 and 130 nm, with eucalyptus extract and between 23 and 39 nm, with peppermint extract. Likewise, their fungicidal action against the fungus *Colletotrichum capsici* that affects chili peppers (between 93.75% and 99.78% against conventional fungicides) was verified (11). On the other hand, CuO NPs sized between 6.93 and 20.70 nm, obtained with *Citrus sinensis* extract, showed a bactericidal capacity against *Escherichia coli* and *Staphylococcus aureus,* besides a non-cytotoxic nature in the mouse fibroblast L929 cell line using the XTT cell viability assay (12).

Complex healthcare contexts have emphasized the production of biocidal materials, such as wound dressings, based on the impregnation of metallic silver nanoparticles in wool, cotton, viscose, nylon, and polyamide (13–15). In a bandage evaluation by impregnation of cotton fabric with crystalline silver nanoparticles and bimetallic Ag/Cu composites, the antimicrobial properties of silver and antifungal properties of bimetallic fabrics were confirmed for a wide range of multidrug-resistant bacteria and fungi, such as *Enterobacter aerogenes, Proteus mirabilis, Klebsiella pneumoniae, Candida albicans* yeasts, and micromycetes (16).

With respect to the type of nanomaterial, although numerous studies have reported the green synthesis of copper oxide nanoparticles using plant extracts, the majority have focused on cupric oxide (CuO) (17–19). However, cuprous oxide (Cu₂O) exhibits distinct physicochemical properties that warrant independent investigation. Unlike CuO, which contains copper in the +2 oxidation state, Cu₂O is composed of Cu^+^ species, conferring different redox behavior, electronic structure, and surface reactivity. These differences influence not only the synthesis mechanisms under green conditions but also the resulting nanoparticle size, morphology, and biological interactions (9, 20, 21). This often results in smaller particle sizes and narrower size distributions compared with CuO nanoparticles synthesized under similar conditions. Furthermore, Cu₂O has been reported to exhibit enhanced antimicrobial activity at lower concentrations, attributed to its higher surface reactivity and controlled release of copper ions (22, 23).

In addition, Cu₂O nanoparticles have demonstrated favorable compatibility with polymeric substrates, making them particularly suitable for textile functionalization (24, 25). Their lower oxidation state and reduced tendency to induce fiber degradation represent a significant advantage for applications involving cotton–polyester blends. For these reasons, the present study focuses on the green synthesis of Cu₂O nanoparticles using *Myrciaria dubia* extract and their incorporation into textile materials, with the aim of evaluating their structural characteristics and antibacterial performance against *Escherichia coli* strains.

*Escherichia coli* has become established as a relevant reservoir of genes encoding extended-spectrum β-lactamases (ESBLs), which limits the use of β-lactam antibiotics and positions it as a key bioindicator of antimicrobial resistance in Gram-negative bacteria. Extended-spectrum β-lactamases are enzymes produced by bacteria, such as *E. coli,* that confer resistance to β-lactam antibiotics, especially penicillins and cephalosporins. Antimicrobial resistance may also arise from chromosomal mutations, broadening the spectrum of affected drugs. ESBL-producing *E. coli* strains represent a major challenge in human and veterinary medicine due to their ability to disseminate resistance genes through horizontal transfer, mainly via plasmids, and to contribute to their spread in the environment. Several resistance genes, including *TEM, SHV*, and *CTX-M*, constitute the main genotypes associated with the multidrug-resistant phenotype in ESBL-producing *E. coli* strains (26, 27).

Extended-spectrum β-lactamase-producing *Escherichia coli* (ESBL-EC) represents a growing public health problem due to its frequent association with additional antimicrobial resistance genes. This co-resistance generates non-wild-type phenotypes against multiple antibiotic classes, which may limit therapeutic options during hospitalization. Several studies have reported that ESBL-EC isolates from foods exhibit elevated minimum inhibitory concentrations and resistance to antimicrobials, such as avilamycin, colistin, quinolones, and fluoroquinolones (28).

The objective of this research was to evaluate and compare the efficacy of cuprous oxide (Cu₂O) nanoparticles synthesized through biogenic and chemical methods, using *Myrciaria dubia* and ascorbic acid as reducing agents, respectively. The study aimed to analyze how the synthesis method influences the physicochemical properties of the nanoparticles, their integration into functionalized textiles, and their antibacterial activity against *Escherichia coli* strains, including ESBL-producing variants, with the goal of exploring their potential in the development of sustainable antimicrobial materials.

## MATERIALS AND METHODS

The reagents necessary for the synthesis of metallic nanoparticles were acquired from Merck Millipore, assuring their purity. Water from the Thermo Scientific system (Barnstead Smart2Pure, Massachusetts, USA) was used in all procedures.

The textile used in the experiments consists of 70% cotton and 30% polyester, with a weft and taffeta configuration. All functionalized and control textile samples were previously sterilized and stored in Kraft paper envelopes and hermetically sealed bags, ensuring their suitability for future use. It is worth mentioning that the applicability of this work is an extension of previous research linked to the functionalization of textiles made by the authors of this manuscript (29–31).

### Synthesis and functionalization of “post-synthesis” textiles with Cu_2_O NPs by the chemical method

For this process, the work carried out by one of the authors in a previous manuscript (29) was taken as a basis with some modifications. It began with the synthesis of Cu_2_O nanoparticles, using only chemical reagents. The functionalization of the fabric (70% cotton/30% polyester) was carried out after the synthesis of the textile material, and this approach was called “post-synthesis.”

The process started with the preparation of the precursor, copper sulfate at 0.05 M, which was kept under stirring at 500 rpm for 10 min at 23°C. Then, 22.5 mL of sodium hydroxide (NaOH) at 7.5 M was added dropwise, maintaining vigorous stirring at 1,200 rpm for approximately 20 min. Next, ascorbic acid at 1.13 M was added dropwise, carrying out the whole procedure at a temperature of 23°C. The resulting mixture was kept under stirring at 1,200 rpm for 10 min. The formation of a colloid with sedimentation was observed and allowed to precipitate for 48 h. Subsequently, the supernatant containing the Cu_2_O nanoparticles was removed because they were characterized by UV-vis.

For the functionalization of the textile, 450 mL of the Cu_2_O nanoparticle colloid was placed in a beaker, subjecting it to magnetic stirring (900 rpm). Immediately after, the textile piece was immersed and kept in this condition for 10 min at 23°C. Subsequently, the textile was removed and allowed to drain for 10 min. Finally, the piece was subjected to a drying process in a forced convection oven for 15 min at 100°C.

### “*In situ*” synthesis and functionalization of textiles with Cu_2_O NPs using bioactive compounds from *M. dubia*

In this study, the synthesis process of Cu_2_O nanoparticles was evaluated using *M. dubia* (camu-camu) juice as a reducing agent, due to its high ascorbic acid content. The functionalization of the textile (70% cotton/30% polyester) was carried out simultaneously with the formation of nanostructures, classifying this approach as “*in situ*” functionalization (29).

The methodology began with the preparation of the precursor, copper sulfate, at 0.05 M, with the mixture stirred at 500 rpm for 10 min at room temperature (~22°C). Then, the textile piece to be functionalized was immersed and kept in these conditions for approximately 3 min. And 22.5 mL of NaOH at 7.5 M was added dropwise, increasing the stirring to 1,200 rpm, followed by the gradual addition of 90 mL of previously filtered *M. dubia* juice. The mixture, together with the textile, was kept stirring at 1,200 rpm for 10 min at room temperature.

Subsequently, the textile was removed and allowed to drain for 10 min, performing a superficial washing with a wash bottle containing distilled water. Then, the textile piece was placed in a forced convection oven at 100°C for 15 min.

### Synthesis and functionalization of “post-synthesis” textiles with Cu_2_O NP bioactive compounds from *M. dubia*

In this context, the synthesis of Cu_2_O nanoparticles was initially carried out by preparing the precursor, copper sulfate at 0.05 M. Subsequently, 22.5 mL of NaOH at 7.5 M was gradually added, followed by the dropwise incorporation of 90 mL of previously filtered *M. dubia* juice. The resulting mixture of nanoparticles was refrigerated for 24 h, and a volume of 500 mL was selectively extracted from the supernatant, which contained the nanoparticulate material.

This extracted colloid was transferred to a beaker, where magnetic stirring at 600 rpm was started. The textile piece (70% cotton/30% polyester) was then immersed and kept stirring at 1,200 rpm for 1 h at room temperature (~22°C). Subsequently, the textile piece was removed and, with the help of a wash bottle with distilled water, a surface wash was performed to eliminate possible agglomerates. Finally, the piece was subjected to a drying process in a forced convection oven at 100°C for 15 min.

### Characterization of nanomaterial and functionalized textiles

The NPs were subjected to initial analysis by UV-vis spectrophotometry (UV 1900, Shimadzu) in the range of 380 to 900 nm. The elemental concentration of copper (Cu) was determined using an Atomic Absorption Spectrophotometer (Agilent Technologies, 200 AA series). To evaluate the size and shape of the NPs, TEM was used, where 5 µL of the colloid was applied on a copper grid (400 mesh) coated with carbon, and the measurements were performed in a JEOL equipment (JEM 2011 model) with a voltage acceleration of 120 kV. The FTIR spectrum was calibrated at a resolution of 4 cm^−1^ and in the transmission mode (4,000–500 cm^−1^) using an FTIR (Nicolet 6700, Thermo Scientific). The spectra were analyzed using OMNIC 8.1 software (Thermo Fisher Scientific).

The present study represents the continuation of previous work carried out by the research team in the field of nanoparticle-functionalized textiles. In this context, the scanning electron microscopy (SEM) images correspond to prior studies conducted by the research group and applied to the investigation of other etiological agents (29–31). Therefore, the considerations adopted for the preparation of samples intended for SEM analysis were as follows: the samples were sputter-coated with a thin layer of gold, with an exposure time of 100 s at 20 mA. Image analyses were performed using a JEOL JSM-6390LV SEM operated at an accelerating voltage of 20 kV and a spot size of 50. Energy-dispersive X-ray measurements were carried out using the SEM equipped with an OXFORD EDS 7582-M system. Additionally, the samples were characterized using a Nicolet iS50 FTIR infrared spectrometer (Thermo Fisher Scientific) in the range of 500 to 4,000 cm⁻¹, with 200 scans per sample.

### Bacterial challenge

*E. coli* BLEE and *E. coli* (ATCC 25922) strains were inoculated in Tryptone Soy Agar (TSA) medium and incubated for 24 h at 37°C, then transplanted and incubated for 24 h at 37°C in Tryptone Soy Broth (TSB) medium.

Cloths with 1 cm × 2 cm Cu_2_O nanoparticles were placed in sterile 50 mL conical tubes with 1 mL TSB of a solution of *E. coli* BLEE and *E. coli* ATCC 25922 at a concentration of 1 × 10^5^ colony forming units (CFU)/mL, approximately, and left to incubate for 24 h at 35°C. Subsequently, 4 mL of fresh TSB solution was added and stirred vigorously at 60 RPM for 1 min to dislodge the microorganisms from the fabric and inoculated with a 1 µL loop in TSA by the duplicate streak seeding method. The plates were incubated for 24 h at 35°C, and readings compared to the negative control (cloths without nanoparticles) were performed.

%Bacterialreduction=100[(B−A)/B]

Where *A* are the CFUs recovered from the challenge of the cloths with nanoparticles, and *B* are the CFUs recovered from the cloths without nanoparticles.

### Statistical analysis

An analysis of variance was performed with *P* < 0.05 and 95% confidence.

## RESULTS AND DISCUSSION

**Fig 1:**
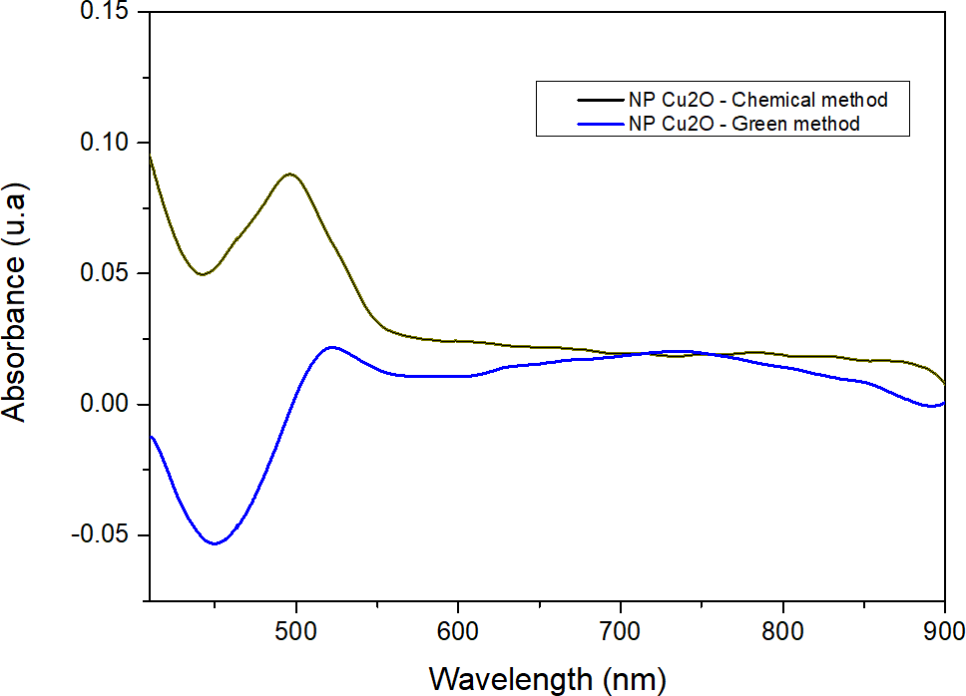
UV-vis spectrophotometry of Cu_2_O nanoparticles synthesized by chemical and green route methods.

Figure 1 shows the UV-vis spectrophotometry results of Cu_2_O NPs synthesized by the chemical and green route methods. They show significant differences between the two methods of cuprous oxide nanoparticle synthesis: the green route using *M. dubia* juice and the conventional chemical method. The chemical synthesis spectrum exhibits a distinct peak at a wavelength of 495 nm, while the green method shows its maximum at 521 nm. This shift toward longer wavelengths in the green method indicates a redshift, suggesting the presence of smaller nanoparticles. The observed redshift in the green route spectrum is consistent with the reduction in nanoparticle size, which can be attributed to the influence of bioactive compounds present in *M. dubia* juice. The interaction between these compounds and the copper precursors may act as an efficient reducing agent, promoting the formation of smaller nanoparticles. On the other hand, it is crucial to note that, despite the differences in size, the chemical method exhibits a markedly higher absorbance compared to the green route. This higher absorbance suggests a higher nanoparticle density or a broader size distribution, which is directly linked to a more significant yield. The higher absorbance in the chemical method may be due to a higher concentration of nanoparticles in the solution, indicating a higher efficiency in terms of synthesis yield. The choice between the two methods is that the smaller the nanoparticles are, the better they can be incorporated into the textile fiber. However, this result supports the viability of both synthesis routes, each with its advantages and potential applications in the field of nanotechnology.

**Fig 2:**
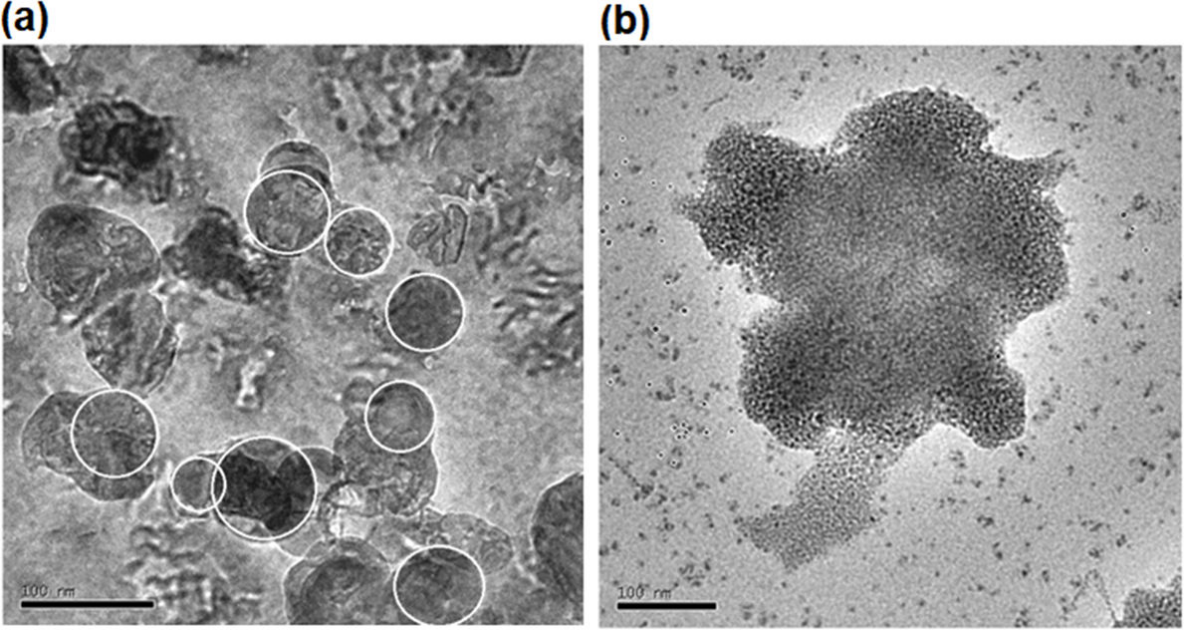
TEM images of Cu_2_O nanoparticles synthesized by (**a**) the chemical method and (**b**) the green method.

The synthesis of cuprous oxide nanoparticles by two different methods, biogenic synthesis and chemical synthesis, was thoroughly analyzed by TEM (Fig. 2). The results revealed significant differences in size and morphology between the nanoparticles obtained by each method. In the case of the biogenic synthesis, which involved the use of *M. dubia* juice as a reducing agent, the cuprous oxide nanoparticles presented an average size of 10 ± 2.22 nm. This biogenic method was characterized by the formation of smaller and more uniform particles, suggesting precise control over nanoparticle nucleation and growth. The morphology observed in TEM images revealed a homogeneous distribution of spherical particles, indicating a well-defined and efficient synthesis.

On the other hand, chemical synthesis, which involved the use of ascorbic acid as a reducing agent, led to cuprous oxide nanoparticles with an average size of 75 ± 2.8 nm. Compared to biogenic synthesis, this chemical method produced larger and less uniform particles. The observed morphology showed variability in nanoparticle shape, with some particles exhibiting irregular shapes. The detailed analysis of TEM images also revealed valuable information about the size distribution of nanoparticles. Analyzing the size distribution, there was a slight difference between the two methods. In the case of the chemical synthesis, the size distribution was more focused on the average value, indicating greater homogeneity in nanoparticle production. In contrast, green synthesis showed a broader distribution, suggesting greater variability in nanoparticle size.

Besides the size and morphology information, transmission electron microscopy allowed the observation of structural details at the nanometer level, such as the crystallinity of nanoparticles. This analysis revealed that the nanoparticles obtained by both methods exhibited a crystalline structure, which supports the quality and purity of the synthesized products.

The larger average size and lower uniformity observed in chemically synthesized Cu₂O nanoparticles, compared with those obtained via the biological route, can be attributed to fundamental differences in the nucleation and growth mechanisms involved in the two processes. In chemical synthesis, the reduction of Cu²^+^ ions occurs rapidly in the presence of a single reducing agent, which often results in a limited number of nucleation sites, followed by uncontrolled crystal growth. In addition, the limited availability of stabilizing species promotes particle aggregation and Ostwald ripening, leading to broader size distributions.

In contrast, biological synthesis using *Myrciaria dubia* extract provides a complex mixture of polyphenols, flavonoids, and organic acids that simultaneously act as reducing, capping, and stabilizing agents. These biomolecules promote a higher density of nucleation events while effectively limiting particle growth through surface adsorption, resulting in smaller and more homogeneous Cu₂O nanoparticles. The presence of multiple functional groups capable of coordinating copper ions further contributes to controlled crystal growth and enhanced colloidal stability.

Accordingly, the differences in nanoparticle size and uniformity observed between the two methods are intrinsically linked to the chemical environment governing the synthesis process. Green synthesis offers superior control over particle growth and dispersion, which is particularly advantageous for applications requiring a uniform distribution of nanoparticles on textile substrates.

**Fig 3:**
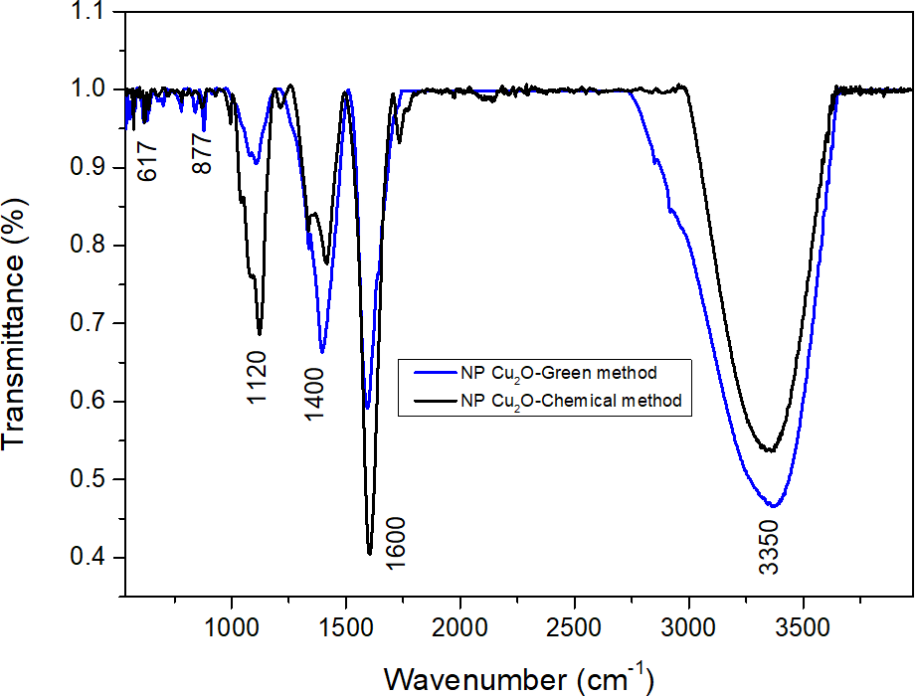
FTIR spectrum of Cu_2_O nanoparticle colloids synthesized by green and chemical routes.

Figure 3 shows the comparative analysis of the FTIR spectra of Cu_2_O NPs obtained by green synthesis and chemical synthesis, revealing subtle but significant differences in the spectroscopic characteristics of these materials. The presence of peaks at 617 and 877 cm^−1^ in both cases, with slightly higher intensity in the green synthesis, suggests the existence of specific bonds related to the Cu_2_O phase. This result indicates that both synthesis methods lead to the formation of the Cu_2_O crystalline phase, corroborating the efficiency of both in obtaining the compound of interest (29, 32). In the region of 1,120 cm^−1^, a much more intense peak is observed in the chemical synthesis than in the green synthesis. This behavior could be related to the presence of specific functional groups associated with the reducing agent used in each method. The differential intensity in this region can provide valuable information about the surface chemical interactions and surface layer characteristics of the nanoparticles. In this case, a fainter signal is observed in the context of green synthesis, suggesting the possibility that the bioactive compounds present in the *M. dubia* juice have undergone a more efficient consumption and accelerated reaction in the NP formation process. This lower signal intensity could be associated with a coating on the NPs, possibly linked to an additional functionalization with the residual components of the synthesis medium (33).

The presence of the signal at 1,400 and 1,600 cm^−1^ in both types of Cu_2_O NPs, which is related to the existence of carboxylate groups, was also found (34). In the context of green synthesis, involving the use of *M. dubia* juice as a reducing agent, it is postulated that bioactive compounds present in the juice, such as organic acids, could contribute to the formation of these carboxylate groups. The possible derivation of these groups from the carboxylic acids inherent in the biological matrix of *M. dubia* is put forward as a plausible hypothesis. The presence of such carboxylate groups on the surface of the NPs could generate propitious anchoring sites for various organic molecules, exerting a possible influence on the physical and chemical properties of the nanoparticles. For the chemical synthesis scenario, which incorporates ascorbic acid as a reducing agent, it is suggested that the presence of carboxylate groups could be linked to the thermal decomposition of ascorbic acid during the synthetic process. The oxidation of ascorbic acid could generate intermediate products containing carboxylate groups, which could adsorb on the surface of Cu_2_O NPs during their formation. This phenomenon would thus contribute to the manifestation of carboxylate groups on the nanoparticles resulting from the chemical synthesis.

Ultimately, the signal intensification at 3,350 cm^−1^ in the green synthesis stands out, suggesting the presence of functional groups linked to biogenic synthesis. In this region, the association of hydroxyl groups, amides, or water groups adsorbed on the surface of the nanoparticles is speculated. The distinctive feature of the green synthesis lies in its propensity to favor the formation of a layer with OH radicals, which is linked to a biofunctionalization process. This particularity is reflected in the spectrum as a more intense signal compared to the case of chemical synthesis (35).

**Fig 4:**
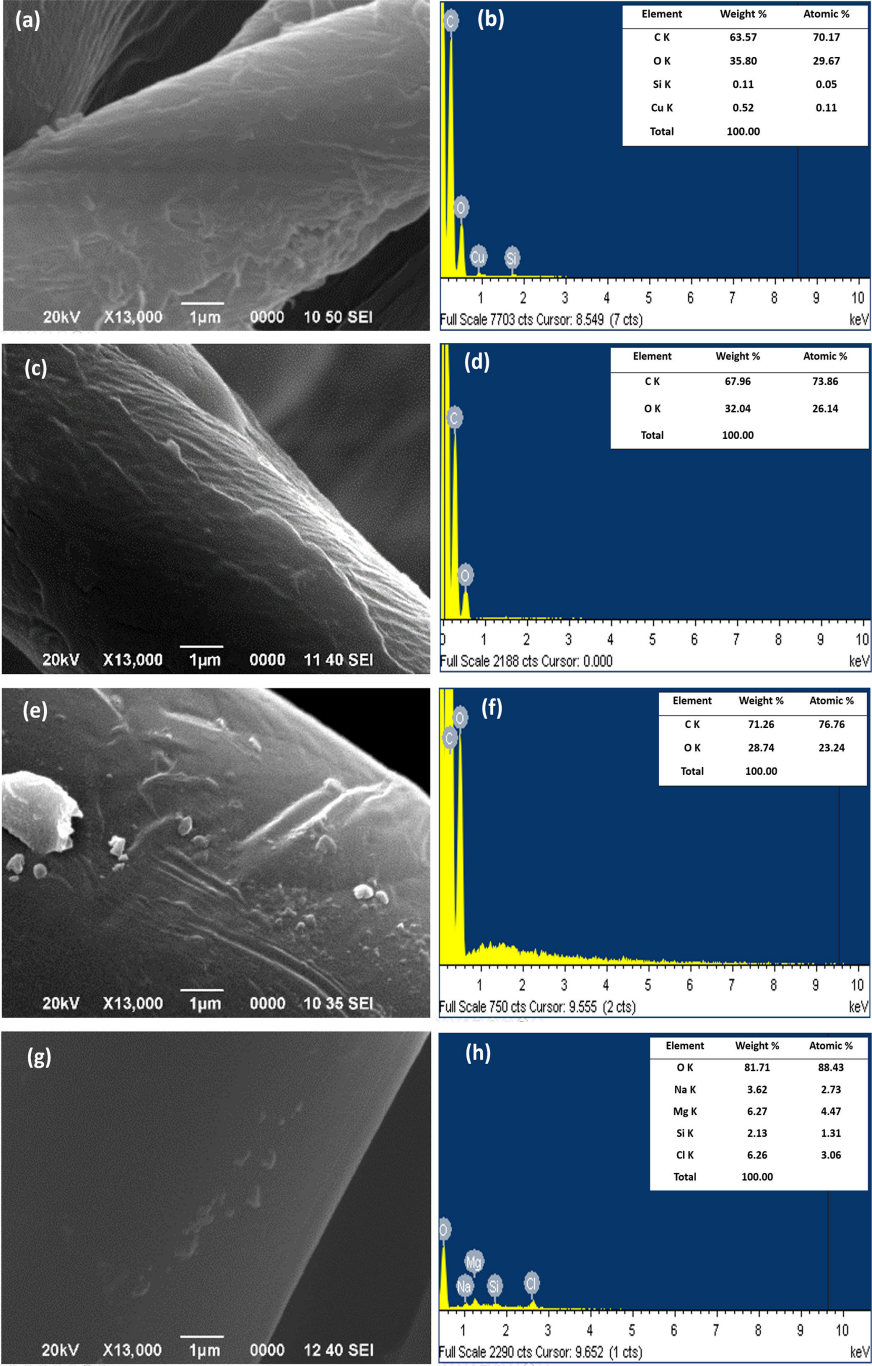
SEM images and EDS elemental characterization of textile fibers functionalized with Cu_2_O NPs. (**a and b**) *In situ M. dubia*, (**c and d**) post-synthesis *M. dubia*, (**e and f**) post-synthesis chemical, and (**g and h**) control textile.

Figure 4 exhibits the results obtained by SEM and elemental analysis by energy dispersive spectroscopy (EDS) of the textile fibers functionalized with Cu_2_O NPs by the green method, *in situ* (a and b) and post-synthesis (c and d), as well as by the post-synthesis (e and f) chemical method, compared to the control textile (g and h). The data reveal that the most effective impregnation method is the one in which the textile fiber is immersed during the process of reduction and formation of the Cu_2_O nanostructure.

In particular, it is highlighted that the *in situ* impregnation methodology using Cu_2_O NPs obtained by the green method shows the significant presence of 0.52% of elemental copper (Cu) in the analyzed sector of the textile fiber, while the other treatment methods do not present specific values. This finding suggests that immersion during the synthesis process favors a more effective adhesion of the nanoparticles to the textile fiber, resulting in a higher presence of detectable elemental Cu. It is worth mentioning that this result reflects the analysis on the surface; however, the very core of the textile fiber was also impregnated. It is essential to highlight that all functionalized textile samples, either by green or chemical synthesis, were treated with Cu_2_O NPs at a constant concentration of 734 ppm. This data provides uniformity in the treatment, allowing more accurate comparisons between the different functionalization methods. These results have been previously reported by one of the authors of this manuscript, so the information is provided in greater detail (29).

**Fig 5:**
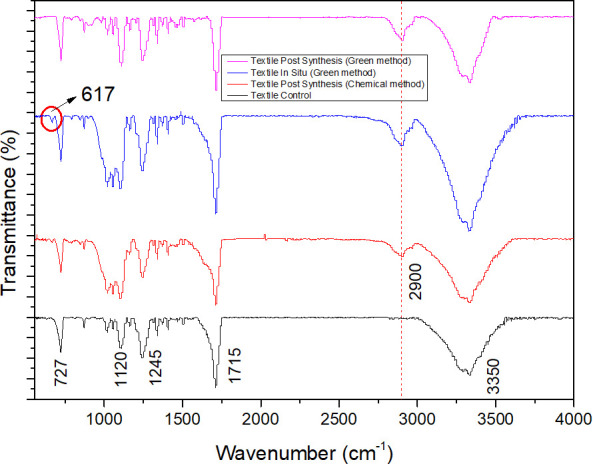
FTIR spectrum of control textile fibers and those functionalized with Cu_2_O NPs under different methods.

The textile was subjected to a detailed analysis by FTIR to discern any variation regarding the control. In this context, Fig. 5 shows the spectra of the textiles treated by the different methods and the Cu_2_O NPs previously described. It is highlighted that the textile treated with the *in situ* method, using Cu_2_O NPs obtained by green synthesis, is the only one that presents a distinctive signal at 617 cm^−1^, thus corroborating the results of the EDS characterization, where the highest elemental copper content (0.52%) was recorded.

On the other hand, an intense signal is observed at 3,350 cm^−1^ in the spectrum of the control textile, related to the vibration of the OH groups of the cellulose present in cotton. For the NP-treated textiles, this signal corresponds to the OH radical, indicating the incorporation of the nanoparticles. In addition, a signal is noted at 2,900 cm^−1^ in the treated textiles, resulting from the –CH interaction of the cellulose with the NPs, suggesting the formation of a more pronounced peak.

Concerning polyester, specific signals are identified in the FTIR spectrum, such as the vibration of the ester groups (–C=O) at 1,715 cm^−1^ and the ester groups at 1,245 cm^−1^. The band at 727 cm^−1^, characteristic of polyester, confirms the presence of this material in the analyzed textiles.

As observed (Table 1), there was a significant difference between the control group and the treatment with copper oxide nanoparticles for *E. coli* ATCC 25922. However, there was no significant difference between the control and the treatment with copper oxide nanoparticles for *E. coli* BLEE. A previous study showed the antibacterial effect of copper oxide nanoparticles on *E. coli* ATCC 8739, although there was no significant antibacterial effect of copper oxide nanoparticles for *E. coli* BLEE (29).

**TABLE 1:** Effect of copper oxide nanoparticles (734.132 ppm) against *E. coli* ATCC 25922 and *E. coli* BLEE^*b*^

	Control fabric (UFC/mL)	Post-synthesis (ascorbic acid)-percentage of inhibition	*In situ* (*M. dubia*)-percentage of inhibition	Post-synthesis (*M. dubia*)-percentage of inhibition	Sig.
*E. coli* ATCC 25922	286 × 10^5^ ± 1,131,370.85^*a*^	4,500 ± 707.11 CFU/mL (99.98%)^*a*^^,^^*c*^	27,500 ± 19,091.88 CFU/mL (99.90%)^*a*^^,^^*c*^	16,000 ± 11,313.71 CFU/mL (99.94%)^*a*^^,^^*c*^	<0.05
*E. coli* BLEE		≥100,000 CFU/mL^*c*^^,b^	≥100,000 CFU/mL^b^	≥100,000 CFU/mL^b^	Not significant

^*a*^ There was a significant statistical difference (*P *< 0.001) between the treatments and the fabric control for *E. coli *ATCC 25922.

^*b*^ No significant statistical difference was shown between treatments and fabric control for *E. coli* BLEE.

^*c*^ A significant statistical difference (*P *< 0.001) was shown between the growth of *E. coli *ATCC 25922 and *E. coli *BLEE.

In this work, the action of copper nanoparticles exposed to *E. coli* was a growth inhibition higher than 99.90% because Cu-NPs produce the formation of cell filaments and subsequent cell death. Filamentation is an abnormal growth of bacteria when cells continue to elongate with multiple chromosomal copies and do not undergo septum formation, resulting in no cell division (36). This process is a primary defense mechanism by the bacterial cell undergoing SOS, such as the stress response. The SOS response exerts a role in the exposure of *E. coli to* agents that injure DNA or interfere with DNA replication, inducing about 30 different proteins for damage tolerance (37, 38). One of the SOS gene products, the SulA protein, encoded by the *sfiA* gene*,* is responsible for filament formation. SulA inhibits cell division by blocking the formation of the bacterial cellular septum-forming ring, composed of FtsZ proteins, and, thus, leads to filament formation. SulA interacted directly with the FtsZ monomer. By sequestering FtsZ, the cell could directly bind DNA damage to inhibit cell division (39, 40). Another detail is that exposure of *E. coli* cells to copper nanoparticles results in the depolarization of the cell membrane. This dissipation of membrane potential is the basis for cell filamentation (41).

Another action of Cu-NPs that may have occurred in *E. coli* is oxidative stress produced by the formation of reactive oxygen species (ROS), leading to cell damage and, consequently, to bacterial killing. Copper nanoparticles act as catalysts in Fenton-type reactions to generate ROS, which are highly reactive free radicals, such as hydroxyl radicals (OH) and superoxide (O_2_^−^) (42). A consequence of this process is lipid peroxidation, which comes to be the oxidative degradation of polyunsaturated lipids in *E. coli* at the plasma membrane level. These changes in fatty acid position modify the physical properties of the lipid bilayer, whose consequence is decreased membrane fluidity, increased membrane leakage, and damage to integral membrane proteins, indirectly regulating which finally leads to toxicity and bacterium death (43, 44). Another cause of *E. coli* growth inhibition by exposure to reactive oxygen species produced by Cu-NPs is that oxidation of proteins occurs at the cellular level, leading to modifications of amino acid side chains and producing alterations of the protein structure, which results in functional changes that disrupt cell metabolism, being a cause of cell death (45). In addition, another cause of the lack of growth of *E. coli* would be again the influence of free radicals, which are reactive oxygen species that damage nitrogenous bases and sugar residues of DNA, producing injuries, such as single and double-strand breaks, and forming, at the same time, compounds of groups of bases and sugars in cross-links with other molecules leading to the inhibition of DNA replication (46).

In contrast, no significant effect of copper nanoparticles on *E. coli* BLEE growth inhibition was observed in this research, which could be due to the relationship between metal and antibiotic resistance in plasmids. Antimicrobial metal resistances are often found in the same mobile gene elements (MGE) as antibiotics (47). Moreover, as referred to by Hobman and Crossman in 2015, enterobacteria present a system that detoxifies them and correctly compartmentalizes copper through load modification and an efflux system. This is achieved by the presence of four chromosomal systems (*cue*, *cus*, *pco, and cop),* considered the bacterial homeostatic protein networks of copper (48).

The lack of inhibition of *E. coli* BLEE growth against copper nanoparticles observed in this work agrees with the above-mentioned reference that the *cue* (copper efflux) system is considered the main mechanism responsible for *E. coli* resistance to copper under both aerobic and anaerobic conditions (49). Thus, copper enters the cell through porins, possibly OmpC and OmpF, and enters the cytoplasm. The *cue* system is constituted by the CopA-type ATPase efflux of the inner membrane that translocates Cu(I) to the periplasm. Then, *CueO,* a multi-copper oxidase, oxidizes Cu(I) to Cu(II); the latter is less toxic. Finally, *CusCFBA* constitutes an efflux pump that expels Cu(II) from the periplasmic to the extracellular space (50). It is noteworthy that in the mechanism under anaerobic and extreme copper stress conditions, *E. coli* uses the *CusCFBA* efflux of the *cus* system, through which the *cusCFBA* operon is transcriptionally regulated in response to stress and elevated Cu(I) levels in the cell envelope by the *Cus*RS sensor-regulatory system (51–53). Meanwhile, resistance to copper at the plasmid level is encoded by the *pco* system, which provides enhanced resistance of *E. coli* to this metal. This *pco* system is encoded by a group of nine, or occasionally, 10 *pco* genes: *pcoGFE*, *pcoABCDRS,* and *pcoE*, which are arranged as two operons, and a separate gene, whose mechanism of action against copper is through copper efflux into the extracellular part (54–56).

About the above-mentioned in this work, *E. coli* BLEE would be showing a cross-resistance, where the resistance system (copper efflux pump) presents resistance to a beta-lactam antibiotic and copper (50).

In this research, copper nanoparticles did not show inhibitory action on the growth of *E. coli* BLEE, and this could be due to a co-resistance mechanism. This occurs when antibiotic and metal resistances are physically located in the same genetic element, such as in a plasmid (50). This was reported by Amachawadi et al. and Hasman and Aarestrup (57, 58), stating that the copper resistance gene, *tcrB*, is horizontally transferable and linked to genes encoding macrolide and glycopeptide resistance (57, 58). The coexistence of the *oqxAB* gene conferring resistance to quaternary ammonium compounds, chlorhexidine, fluoroquinolones, nalidixic acid, ciprofloxacin, quinolones, and beta-lactams (*bla*_CTX-M_), and the copper (*pco*) and silver (*sil*) resistance operons on the same plasmids has also been reported in *E. coli* (59–61).

Understanding the genetic linkage of resistance genes, including their association with different MGEs, such as integrons and transposons, is critical to fully understand the risks of horizontal transfer of antibiotic and metal resistance genes between bacteria (62, 63).

### Conclusions

This study demonstrates that the synthesis of cuprous oxide (Cu₂O) nanoparticles through biogenic and chemical methods yields differentiated outcomes in terms of size, morphology, and antimicrobial efficacy when applied to functionalized textiles. The biogenic synthesis, employing *Myrciaria dubia* as a reducing agent, resulted in smaller, more uniform nanoparticles, which facilitated better integration with textile fibers and exhibited notable antibacterial activity against *Escherichia coli* ATCC 25922. In contrast, although the chemical method produced larger nanoparticles, its antibacterial effectiveness remained significant, highlighting that both approaches offer specific advantages depending on the intended application. Nevertheless, the lack of activity against ESBL-producing strains suggests the need to explore more complex bacterial resistance mechanisms and potential synergies with other antimicrobial agents. Overall, these findings underscore the potential of green synthesis as a sustainable alternative for the development of bioactive textiles, contributing to innovation in the field of antimicrobial materials and applied nanotechnology.

## Data Availability

The original data are available upon request from the corresponding author.
